# The Association between Postload Plasma Glucose Levels and 38-Year Mortality Risk of Coronary Heart Disease: The Prospective NHLBI Twin Study

**DOI:** 10.1371/journal.pone.0069332

**Published:** 2013-07-19

**Authors:** Jun Dai, Ruth E. Krasnow, Lei Liu, Stephen G. Sawada, Terry Reed

**Affiliations:** 1 Division of Epidemiology, Department of Medicine, Vanderbilt University Medical Center, Nashville, Tennessee, United States of America; 2 Department of Applied Health Science and the Department of Epidemiology and Biostatistics, School of Public Health - Bloomington, Indiana University, Bloomington, Indiana, United States of America; 3 Indiana Center for Vascular Biology and Medicine, Indianapolis, Indiana, United States of America; 4 Center for Health Sciences, SRI International, Menlo Park, California, United States of America; 5 Department of Preventive Medicine, Feinberg School of Medicine, Northwestern University, Chicago, Illinois, United States of America; 6 Krannert Institute of Cardiology, Department of Medicine, Indiana University Medical Center, Indianapolis, Indiana, United States of America; 7 Department of Medical and Molecular Genetics, Indiana University School of Medicine, Indianapolis, Indiana, United States of America; University Heart Center, Germany

## Abstract

**Background:**

Due to the paucity of direct evidence, we aimed to evaluate whether the association between postload plasma glucose levels (ppGlucose) and long-term risk of mortality from coronary heart disease was independent of or attributable to genes and common environment.

**Methods and Findings:**

From the prospective National Heart, Lung, and Blood Institute (NHLBI) Twin Study, we included 903 middle-aged male twins, who were nondiabetic, free of coronary heart disease at baseline (1969–1973), and followed for up to 38 years for coronary heart, cardiovascular, and all-cause mortality. Frailty survival models were used to estimate hazard ratio (HR) for various associations: overall (equivalent to singleton population association), within-pair (controlling for genes and environment common to co-twins), and between-pair association (reflecting influences of common factors). Overall associations were statistically significant for coronary heart and cardiovascular but not all-cause deaths after controlling for known risk factors. The associations were not statistically significant in within-pair analyses. The within-pair association was not statistically different by zygosity for specific and all-cause death risk. After the full adjustment for known risk factors, HR (95% confidence interval) for within-pair association was 1.07 (0.90, 1.28), 1.06 (0.94, 1.19), and 0.99 (0.94, 1.05) for coronary heart, cardiovascular, and all-cause mortality, respectively. The fully adjusted between-pair associations were statistically significant for specific and all-cause death risk: a 50 mg/dL increase in the mean value of ppGlucose for a twin pair was associated with a raised death risk [HR (95% confidence interval) 1.15 (1.02, 1.30), 1.10 (1.02, 1.20), and 1.05 (1.01, 1.09) for coronary heart, cardiovascular, and all-cause mortality, respectively]. Between-pair association was significant in dizygotic but not in monozygotic twins.

**Conclusion:**

The positive association between ppGlucose and long-term coronary heart mortality risk is largely explained by factors shared between co-twins, including familial factors; however, within-pair effects cannot be absolutely excluded.

## Introduction

Hyperglycemia has been considered as a risk factor for cardiovascular events and postload hyperglycemia may be a stronger predictor of cardiovascular outcomes than fasting hyperglycemia [1], [2], [3], [4], [5], [6]. Genetic and environmental factors can influence hyperglycemia [7]. Changes in common environment, rather than genetic factors, may be a more sensible explanation for the national changes in the prevalence and incidence of diabetes [8]. Twin migration studies have illustrated that changes in common environment shared between co-twins alter the risk for cardiovascular disease [9] [10]. Genetic and common environmental factors could influence hyperglycemia-related cardiovascular outcomes, and might alternatively explain why significant improvement in glucose control did not necessarily prevent or retard diabetes-mediated macrovascular disease including coronary heart disease [11]. However, these genetic and common environmental factors have not been well-controlled for or evaluated in previous traditional observational prospective studies [1], [4], [5], [6]. It remains unknown whether the association between postload hyperglycemia and coronary heart disease is attributable to or independent of genes and common environment.

A co-twin design can dissect the association between exposure and outcomes from genes and environment shared between co-twins of a twin pair. Co-twins share germline (genes and inherited epigenetic modifications) and numerous environmental factors, and are naturally matched for these factors. Environmental factors common to co-twins, common environment, can exist throughout their whole life course or in certain time periods of life, and can be both within and outside of the family. Common familial factors include dietary, behavioral, and parental demographic and socioeconomic factors that are shared between co-twins as they grow up and can have a life-long influence, such as the family history of coronary heart disease. Co-twins are also exposed to similar social and cultural environments from other than familial sources, such as food supply/accessibility/processing/service/retail settings, recreational facilities, school, and workplaces. For example, co-twins of a pair in the Southern US share numerous unknown and unmeasured environmental factors that are not shared by those in the Northern US.

For this study, using a unique co-twin design, we primarily examined the prospective association between postload plasma glucose levels (ppGlucose) and the long-term risk of death due to coronary heart disease independent of influences of genes and common environment, and whether these shared factors contributed to the association. We further tested the association for the risk of mortality from cardiovascular diseases and all causes as secondary outcomes.

## Materials and Methods

### Ethics Statement

The National Heart, Lung, and Blood Institute (NHLBI) Twin Study was approved by the Institutional Review Board at each examination site, and all twins gave written informed consent. This study was approved by the Institutional Review Board at Indiana University and Vanderbilt University.

### Study Population

The NHLBI Twin Study, a longitudinal cohort study, was originally designed for the elucidation of genetic and environmental factors in cardiovascular diseases in United States of America. [12]. Initiated in 1969, the NHLBI Twin Study enrolled 514 middle-aged, white male veteran twin pairs (1028 men, 254 monozygotic and 260 dizygotic twin pairs [12]) from the National Academy of Sciences-National Research Council Veteran Twin Registry, who lived within 200 miles of five research centers: Framingham, Massachusetts; San Francisco, California; Davis, California; Los Angeles, California; and Indianapolis, Indiana [13]. The twins were born between 1917 and 1927, and were 42–55 years of age at the baseline examination (exam 1, 1969–1973) [14]. All twins were physically examined at baseline and during follow-up using the well-established Framingham Heart Study protocol to ensure the uniform examination of all the twins by experienced cardiovascular epidemiologists [15]. Zygosity was ascertained by eight red blood cell antigen groups (serotyping 22 erythrocyte antigens) in the 1960s and variable number of tandem repeat DNA markers in the 1980s [12]. In this study, exclusion criteria included missing data on ppGlucose, current use of insulin or oral hypoglycemic agents, ppGlucose >250 mg/dL as previously published [16], or having coronary heart disease diagnosed by the physician at baseline. A total of 23 twins had missing data on ppGlucose; 55 twins were currently using insulin or oral hypoglycemic agents, or had ppGlucose >250 mg/dL; and 61 twins had baseline coronary heart disease diagnosed by the physician. In total 125 twins met one or more exclusion criteria.

### Baseline Measurement

Data on all major cardiovascular risk factors were recorded on a questionnaire through an in-person interview and physical examination, including demographic, socioeconomic, lifestyle, familial, anthropometric, biochemical, and clinical factors. Data on age, years of education, marital status, and smoking status (current smoker, past smoker, and never smoked) were collected. Weight and height were measured. Systolic and diastolic blood pressures were measured using a standard mercury sphygmomanometer [16]. A minimum 9-hour overnight fasting blood sample was drawn to measure levels of triglycerides; total cholesterol; and cholesterol in high-density and low-density lipoproteins using the North American Lipid Research Clinics methodology [16]. Plasma glucose levels at 1-hour after a 50-gram glucose load (ppGlucose) were measured. Diabetic subjects who were diagnosed before the baseline exam did not receive the glucose load based on the research protocol. Baseline diabetes was defined by current use of insulin or oral hypoglycemic agents, or ppGlucose >250 mg/dL [16]. A 12-lead electrocardiogram was recorded, and information on current use of medications was collected. Subjects were interviewed by a physician who completed a medical history questionnaire that included questions about cardiovascular events and procedures [14]. Heart disease and other cardiovascular disease were diagnosed by the physician at baseline [14].

### Assessment of Endpoints and Follow-up

Vital status as well as the cause and date of death through December 31, 2007 were ascertained from medical records in four follow-up examinations (exam 2, 1981–1982; exam 3, 1986–1987; exam 4, 1995–1997; and exam 5, 1999–2000), and through death certificates or the National Death Index [14]. Criteria used for ascertaining outcomes in four follow-up examinations were standardized, and decisions regarding disease diagnosis were made by a panel of investigators: at examinations 2 and 3 two independent physicians reviewed medical records; at examinations 4 and 5 one physician reviewed medical records [14]. Physicians assigned corresponding International Classification of Diseases, Ninth Revision (ICD-9) codes. Death certificates or the National Death Index (ICD-9 codes) were obtained for decedents. The primary endpoint was death from coronary heart disease (410–414). Secondary endpoints were death from cardiovascular diseases (390–398, 402, 404, 410–438) and all causes. Subjects were considered lost to follow-up if a death certificate or coding from the National Death Index could not be traced. Nineteen twins out of the 903 twins (2%) were lost to follow-up due to lack of social security number for tracing a death certificate or the National Death Index. These twins were included in this study and were treated as if they were alive at the date of the end of the study. The lost-to-follow-up twins were similar to other twins except that they were more likely to be currently married and were less likely to be on antihypertensives. The follow up was terminated at the date of death, the end of follow-up, or the loss to follow-up, whichever occurred first.

### Statistical Analyses

The broad sense heritability of ppGlucose was estimated with software package Mx32 using A (additive genetic influence) D (dominant genetic influence) E (unique environment) structural equation modeling [17]. We used a frailty survival model with a Weibull-distributed failure time and a frailty to evaluate the associations [18]. The frailty was a random effect to account for clustering within a twin pair [18]. The ppGlucose was analyzed as a continuous variable. We first adjusted for age at baseline. As performed in previous studies [4], [5], [6] we controlled for known risk factors (other potential confounders) at baseline including: years of education, marital status, smoking status, body mass index, systolic blood pressure, low-density lipoprotein cholesterol, ratio of high-density lipoprotein cholesterol to triglyceride, and use of antihypertensives. As very few twins were on anti-cholesterol agents (0.3%) or anticoagulants (0.9%) at baseline (1969–1973), we did not include the use of these types of medications in the model.

We followed the modeling strategy for twin data described by Carlin et al. [19], [20], [21], [22]. We evaluated the overall/individual effect by treating twins as individuals but accounting for the twin pair clustering through a random effect “frailty”. This analysis was equivalent to testing the association in a singleton population to see whether the previous findings [1], [4], [5], [6] could be replicated in our cohort. Next, we did the co-twin analysis (a matched analysis) by partitioning overall effects into within- and between-pair effects [19]. The within-pair effect was parameterized as the deviation of twin’s ppGlucose from the mean ppGlucose of his twin pair. The between-pair effect was parameterized as the mean ppGlucose of the twin pair. The within-pair association controlled for confounding from genes and common environment. Since monozygotic co-twins share 100% genes while dizygotic co-twins, on average, share 50% of the segregating genes, any difference within a monozygotic twin pair should be caused by environmental factors. Between-pair effects were a combined influence of genes and environment shared between co-twins, in which genetic influence cannot be distinguished from common environmental influences. Detailed explanation of between-pair effects is described in **Text S1**.

A statistical approach to ascertain the genetic role in the association was to test the interaction of zygosity with within-pair effects. If within-pair associations were statistically significantly different by zygosity, the genetic role was important in the association; otherwise, genes were not importantly involved although the existence of a genetic role could not be completely excluded [23].

For practical use, we estimated the relative risk of death for a 50 mg/dL (2.78 mmol/L) increment in ppGlucose. As missing data on covariates were less than 2.2%, we used multiple imputations to account for missing data and obtained fully adjusted parameter estimates. All analyses were conducted using SAS software version 9.2 (SAS Institute). Significance levels were set at *P* = 0.05 (2 sided).

## Results

### Sample Characteristics at Baseline and Cohort Follow-up

There were 903 twins (196 monozygotic and 208 dizygotic twin pairs, 48 unpaired monozygotic twins and 47 unpaired dizygotic twins) included in this study. We excluded 125 twin participants from the original cohort. Excluded twins were similar to those who were included in age, years of education, smoking and marital status. However, they had higher body mass index, systolic blood pressure, ppGlucose, low-density lipoprotein cholesterol, lower ratio of high-density lipoprotein cholesterol to triglyceride, and were more likely to use antihypertensives than retained twins.

The mean age of twins at baseline was 47.8 (range 42–55) years. The level of traditional risk factors was simply expressed by the quintile of ppGlucose in **Table 1**. Twins with higher ppGlucose were more likely to be non-smokers and users of antihypertensives; had higher body mass index, systolic blood pressure, and fasting triglyceride levels; and had lower ratio of high-density lipoprotein cholesterol to triglyceride (*P*<0.05 for all trend tests) (**Table 1**). Within-pair difference in ppGlucose ranged from 0 to 8.7 mmol/L, while between-pair difference ranged from 4.0 to 18.6 mmol/L.

**Table 1:** Characteristics of twins at baseline and summary of follow-up outcomes according to the quintile of postload glucose levels^a^.

Variable		Quintile of Postload Plasma Levels of Glucose (mmol/L)					
	Entire Cohort(N = 903)	[50, 112](N = 181)	[113, 136](N = 180)	[137, 156](N = 177)	[158, 185](N = 183)	[186, 250](N = 182)	*p* value^b^
**Baseline characteristics**							
Postload glucose (mmol/L)	8.3 (2.3)	5.2 (0.78)	6.9 (0.39)	8.2 (0.44)	9.6 (0.4)	11.7 (0.94)	<.001
Age (y)	47.7 (3.1)	47.5 (3.4)	47.5 (3.1)	47.7 (3.2)	48.1 (2.9)	48.4 (3.0)	.35
Smoking (n,%)							.047
Never smokers	377 (41.7)	72 (39.8)	70 (38.9)	72 (40.7)	76 (41.5)	87 (47.8)	
Former smokers	82 (9.1)	13 (7.2)	19 (10.6)	17 (9.6)	10 (5.5)	23 (12.6)	
Current smokers	444 (49.2)	96 (53.0)	91 (50.6)	88 (49.7)	97 (53.0)	72 (39.6)	
Marital status (n, %)							.81
Never married	50 (5.6)	7 (3.9)	14 (7.9)	10 (5.7)	10 (5.5)	9 (5.0)	
Not married currently	52 (5.8)	7 (3.9)	15 (8.5)	12 (6.8)	10 (5.5)	8 (4.4)	
Married currently	794 (88.6)	166 (92.2)	148 (83.6)	154 (87.5)	162 (89.0)	164 (90.6)	
Education (y)	13.1 (2.9)	13.2 (2.9)	13.1 (3.0)	12.7 (2.9)	13.1 (2.9)	13.3 (2.8)	.47
Body mass index (kg/m^2^)	25.6 (3.1)	25.1 (2.7)	25.7 (2.8)	25.4 (2.9)	25.8 (3.4)	26.2 (3.7)	.001
Systolic blood pressure (mm Hg)	127 (16)	121 (14)	125 (15)	127 (17)	129 (15)	132 (18)	<.001
Low-density lipoprotein cholesterol (mmol/L)	3.70 (0.9)	3.78 (0.9)	3.72 (0.8)	3.70 (0.9)	3.62 (0.9)	3.72 (1.0)	.19
High-density lipoprotein cholesterol (mmol/L)	1.19 (0.4)	1.16 (0.4)	1.14 (0.3)	1.19 (0.4)	1.19 (0.4)	1.21 (0.4)	.60
Triglyceride (mmol/L)	1.21 (0.85–1.75)	1.07 (0.81–1.48)	1.12 (0.88–1.60)	1.26 (0.88–1.82)	1.26 (0.85–1.77)	1.38 (0.88–2.25)	.001
High-density lipoprotein cholesterol/triglyceride	0.41 (0.26–0.64)	0.46 (0.31–0.64)	0.42 (0.27–0.63)	0.39 (0.25–0.63)	0.37 (0.26–0.64)	0.35 (0.20–0.70)	.02
Use of antihypertensives	48 (5.3)	3 (1.7)	13 (7.2)	7 (4.0)	9 (4.9)	16 (8.8)	.014
**Summary of follow-up outcomes**							
Person-years (y)	26309	5419	5403	5094	5437	4957	
No. of deaths							
Coronary heart disease	93	7 (7.5)	15 (16)	27 (29)	19 (20)	25 (27)	.0013
Cardiovascular disease	172	22 (13)	27 (16)	39 (23)	38 (22)	46 (27)	<.001
All causes	588	112 (19)	113 (19)	112 (19)	121 (21)	130 (22)	.013

a Values are means (standard deviation) for continuous variables, n (%) for categorical variables, or median (25^th^–75^th^ percentile) for skewed-distributed variables unless otherwise indicated.

b Test for trend across the quintile of postload glucose levels at 1-hour after a 50-gram glucose load. All *p* values are corrected for clustering within a twin pair according to the twin type using linear mixed models for continuous variables, and generalized estimating equation logistic models for dichotomous variables, and repeated proportional odds model with generalized estimating equation for the 3-level smoking, and marital status variables. Raw values for continuous variables are presented.

During the 26,309 person-years of follow-up (median 32, interquartile 24–36, maximum 38 years), 93, 172, and 588 twin subjects died from coronary heart disease, cardiovascular disease, and all causes, respectively (**Table 1**).

### Overall Associations

**Table 2** shows that greater ppGlucose was significantly associated with higher risk of death from coronary heart disease, cardiovascular disease, and all-causes in the whole cohort before and after adjusting for age. After further adjusting for smoking, years of education, marital status, systolic blood pressure, low-density lipoprotein cholesterol levels, ratio of high-density lipoprotein to triglyceride, and use of antihypertensives, the association was attenuated but remained significant for specific-cause deaths. The association became marginally significant for all-cause deaths. For a 50 mg/dL increment in ppGlucose, hazard ratio was 1.13 [95% confidence interval (CI) 1.01 to1.27], 1.09 (95% CI 1.01 to 1.18), and 1.04 (95% CI 1.00 to 1.07) for coronary, cardiovascular, and all-cause deaths, respectively (**Table 2**). The broad sense heritability of ppGlucose was 64.9%. The intraclass correlation coefficient of ppGlucose was 0.69 for monozygotic and 0.14 for dizygotic twins, suggesting a dominant genetic influence on ppGlucose. Due to potential genetic and common environmental confounding for the overall association, we partitioned the overall effect into within-pair and between-pair effects.

**Table 2:** Estimated hazard ratios for death from specific and all causes among twins free of baseline diabetes and coronary heart disease^a^.

Association	Hazard Ratio (95% CI) for Death					
	Monozygotic and dizygotic twins (N = 903)	*P* Value	Monozygotic Twins (N = 440; 196 pairs, 48 unpaired twins)	*P* Value	Dizygotic Twins (N = 463; 208 pairs, 47 unpaired twins)	*P* Value
**Overall Association**						
**Coronary Heart Disease (No. of deaths = 93∶43 monozygotic and 50 dizygotic twins)**						
Crude	1.21 (1.07, 1.36)	.002	1.10 (0.94, 1.29)	.22	1.32 (1.11,1.58)	.002
Age-adjusted	1.19 (1.0, 1.34)	.004	1.10 (0.94, 1.29)	.22	1.28 (1.07,1.52 )	.006
Fully adjusted	1.13 (1.01, 1.27)	.04	1.08 (0.93, 1.26)	.32	1.19 (1.003, 1.40)	.047
**Cardiovascular disease (No. of deaths = 172∶83 monozygotic and 89 dizygotic twins)**						
Crude	1.15 (1.06, 1.25)	<.001	1.07 (0.95, 1.20 )	.30	1.22 (1.10,1.36)	<.001
Age-adjusted	1.13 (1.05, 1.22)	.002	1.07 (0.95, 1.20 )	.30	1.19 (1.07, 1.32 )	<.001
Fully adjusted	1.09 (1.01, 1.18)	.02	1.03 (0.92, 1.16 )	.59	1.15 (1.04, 1.27)	.005
**All-Cause Death (No. of deaths = 588∶272 monozygotic and 316 dizygotic twins)**						
Crude	1.06 (1.02, 1.10)	.005	1.02 (0.97, 1.07)	.50	1.10 (1.04, 1.16)	<.001
Age-adjusted	1.04 (1.00, 1.08)	.048	1.007 (0.96, 1.06)	.78	1.07 (1.02, 1.13)	<.001
Fully adjusted	1.04 (1.00, 1.07)	.053	1.004 (0.96, 1.06 )	.87	1.07 (1.02, 1.13 )	.02
**Within-Pair Association**						
**Coronary Heart Disease (No. of deaths = 93∶43 monozygotic and 50 dizygotic twins)**						
Crude	1.09 (0.91,1.30)	.35	1.06 (0.79,1.43)	.70	1.14 (0.92,1.42)	.24
Age-adjusted	1.08 (0.91,1.29)	.36	1.06 (0.78,1.43)	.71	1.13 (0.91,1.40)	.26
Fully adjusted	1.07 (0.90, 1.28)	.43	1.07 (0.80,1.42)	.64	1.08 (0.92,1.27)	.32
Test for interaction with zygosity^b^		.83				
**Cardiovascular disease (No. of deaths = 172∶83 monozygotic and 89 dizygotic twins)**						
Crude	1.08 (0.96,1.22)	.21	1.00 (0.81,1.25)	.98	1.12 (0.98,1.29)	.10
Age-adjusted	1.07 (0.95,1.21)	.24	0.99 (0.79,1.23)	.92	1.12 (0.97,1.28)	.12
Fully adjusted	1. 06 (0.94, 1.19)	.33	0.96 (0.78,1.19)	.71	1.11 (0.97,1.27)	.13
Test for interaction with zygosity^b^		.72				
**All-Cause Death (No. of deaths = 588∶272 monozygotic and 316 dizygotic twins)**						
Crude	1.01 (0.95, 1.07)	.85	0.97 (0.87,1.08)	.56	1.03 (0.96,1.11)	.42
Age-adjusted	1.00 (0.94, 1.06)	.98	0.96 (0.86,1.07)	.45	1.03 (0.96,1.10)	.48
Fully adjusted	0.99 (0.94, 1.05)	.83	0.97 (0.87,1.07)	.52	1.01 (0.94,1.08)	.84
Test for interaction with zygosity^b^		.80				
**Between-pair Association**						
**Coronary Heart Disease (No. of deaths = 93∶43 monozygotic and 50 dizygotic twins)**						
Crude	1.25 (1.10, 1.42)	<.001	1.11 (0.95,1.30)	.19	1.42 (1.17,1.73)	<.001
Age-adjusted	1.23 (1.09, 1.40)	.0011	1.11 (0.94,1.30)	.21	1.37 (1.13,1.65)	<.001
Fully adjusted	1.15 (1.02, 1.30)	.03	1.09 (0.87,1.37)	.45	1.24 (1.03,1.50)	.02
**Cardiovascular disease (No. of deaths = 172∶83 monozygotic and 89 dizygotic twins)**						
Crude	1.18 (1.08, 1.29)	<.001	1.09 (0.96,1.23)	.19	1.28 (1.13,1.45)	<.001
Age-adjusted	1.15 (1.06, 1.26)	.0011	1.08 (0.95,1.22)	.22	1.23 (1.09,1.38)	<.001
Fully adjusted	1.10 (1.02, 1.20)	.02	1.04 (0.93,1.17)	.47	1.18 (1.05,1.32)	.004
**All-Cause Death (No. of deaths = 588∶272 monozygotic and 316 dizygotic twins)**						
Crude	1.08 (1.03, 1.12)	<.001	1.03 (0.97,1.08)	.42	1.14 (1.07,1.21)	<.001
Age-adjusted	1.05 (1.01, 1.09)	.011	1.01 (0.96,1.07)	.60	1.10 (1.04,1.17)	.001
Fully adjusted	1.05 (1.01, 1.09)	.01	1.01 (0.96,1.06)	.71	1.11 (1.05,1.18)	<.001

a Hazard ratios and 95% confidence interval were estimated for per 50 mg/dL increment in postload glucose levels at 1 hour after a 50 gram glucose load for the overall and the between-pair effects; or per 50 mg/dL difference between co-twins for the within-pair effects. Frailty survival models were used to obtain the estimates to account for clustering within a pair. Fully adjusted hazard ratios were controlled for age (continuous), smoking (never, past smokers, and current smokers), marital status (never, not married currently, and married currently), years of education (continuous), body mass index (continuous), systolic blood pressure (continuous), low-density lipoprotein cholesterol (continuous), ratio of high density lipoprotein cholesterol to triglyceride (continuous), and antihypertensives (yes/no).

b Interaction between within-pair effects and zygosity was tested in the fully adjusted model.

### Within-pair Associations

The within-pair association controlled for genes and common environment. The within-pair association of ppGlucose with coronary, cardiovascular and all-cause deaths was not significant before or after adjustment in the whole cohort or by zygosity (**Table 2**). The interaction between within-pair effects and zygosity was not statistically significant for any specific or all-cause deaths (**Table 2**).

### Between-pair Associations

In **Table 2**, the crude, age-adjusted, and fully-adjusted between-pair association regarding ppGlucose was significant for specific and all-cause death risk in the whole cohort and in dizygotic twins, although adjustment attenuated the association. After the full adjustment, for a 50 mg/dL increment in the pair mean of ppGlucose, hazard ratio was 1.15 (95% CI 1.02 to1.30), 1.10 (95% CI 1.02 to 1.20), and 1.05 (95% CI 1.01 to1.09) for coronary, cardiovascular, and all-cause deaths, respectively (**Table 2**).

Using 20-year and 30-year follow-up data, similar association trends were observed (**Table S1**). Additional adjustment for the family history of coronary heart disease generated similar results (data not shown). We obtained similar results with exclusion of the 19 loss-to-follow-up twins (data not shown). Inclusion of twins with ppGlucose >250 mg/dL, diabetes, or previous coronary heart disease also generated similar results (data not shown). Similar trends were found after total caloric intake, ratio of dietary polyunsaturated to saturated fatty acids, or ratio of dietary monounsaturated to saturated fatty acids was additionally controlled for among a subsample where baseline dietary data were available (data not shown).

## Discussion

In this white male twin cohort followed up to 38 years, we found a positive overall and between-pair association of ppGlucose with the risk of death from coronary heart disease, cardiovascular diseases, and all-cause deaths. Within-pair associations for deaths from specific and all causes were not statistically significant, implying that the overall associations are confounded with genes and common environment. Significant between-pair effects suggested an important role of common factors (genes and environment) in these associations. Between-pair association was significant in dizygotic but not in monozygotic twins. The statistically non-significant interaction between within-pair effects and zygosity suggests that genes are less likely to be important in the association.

### Explanation of the Association between the ppGlucose and Coronary Heart Disease

Our overall associations, equivalent to those found in singleton populations, are roughly consistent with previous general population studies [1], [4], [5], [6], in particular, the three previous prospective non-twin non-family studies which also used a 50-gram glucose load [4], [5], [6]. Our findings are consistent with the latter three studies for death risk from coronary heart disease; and with the two Chicago studies [4], [5] for cardiovascular and all-cause death risk. For coronary heart disease, the fully adjusted overall association in our study is similar to that from a meta-analysis of fifteen cohort studies in Western countries, in which subjects were free of diagnosed diabetes and had ppGlucose measured at baseline [1]. Although for the most part we replicated the previous findings from general populations [1], [4], [5], [6], our study generated evidence over a longer period of follow-up.

To our knowledge, our twin study is the first to elucidate whether the association of ppGlucose with specific and all-cause mortality was free of influences from all genetic and environmental factors shared between co-twins. We controlled for potential known environmental confounders similar to those in the previous studies [1], [4], [5], [6]. Of more importance, we used the co-twin design to additionally control for numerous unknown and unmeasured factors shared by co-twins that could not be controlled for in the previous studies. The within-pair association among monozygotic twins, which removes all such shared confounding factors [19], was not significant. In contrast, between-pair effects as the surrogate of the mixed influence of genes and environment common to co-twins were significant; the overall association observed in our study is largely explained by these shared factors between co-twins. However, we cannot completely eliminate the existence of within-pair effects. For example, for coronary heart outcome, from the entire cohort, the fully adjusted 95% HR confidence interval of the within-pair effect point estimate of 1.07 was (0.90, 1.28); thus, we could not rule out an effect as large as a 10% reduction in hazard or a 28% increase in hazard in relation to a 50 mg/dL within-pair difference in ppGlucose.

Are the shared factors between co-twins confounders or mediators in the association? The within-pair and between-pair model itself cannot distinguish between a confounder and a mediator, as they are statistically identical and only conceptually different [24]. Conceptually, the mediation is understood in the way that “the independent variable causes the mediator, which, in turn causes the dependent variable” [24]. In Carlin et al.’s seminal paper, the between-pair effect was conceptualized as a confounder [19]. Therefore, the shared factors indicated by between-pair effects acted as positive confounders so that the overall association was significant.

It was noticed that the between-pair effects were materially greater among DZ than MZ and significant only in DZ twins in our study. The between-pair effect biologically differs in the influence of genes shared between co-twins, which are 50% genes shared in DZ on average while 100% genes are shared in MZ. Although the between-pair effect is the mixed influence of genes and environment shared between co-twins, Carlin et al. suggested that it might be unwise to invest too much meaning in between-pair effects without detailed explanations [19]. Therefore, it would be debatable to explain that the difference in between-pair effects by zygosity is due to the role of shared genes. Although the twin study assumes that common environment has the same impact on monozygotic and dizygotic twins, the equal environment assumption is highly likely to be violated [25], [26]. Thus, identification of factors that are uniquely common to dizygotic but not monozygotic twins may help specifying common factors, including environment and genes shared between co-twins. It is not unreasonable to start with familial factors [27].

### Potential Mechanisms

The mechanism through which common factors associated with ppGlucose led to survival from specific- and all-cause death is unknown. To our knowledge, evidence exists on a few genetic variants in relation to long-term survival from coronary heart disease and cardiovascular disease among patients with these diseases, such as variants in the gene coding for adenosine monophosphate (AMP) deaminase 1 (AMPD1) [28] and paraoxonase 1 [29]. Variation in the gene for muscle-specific AMPD1 is associated with insulin clearance and thus can influence glucose homeostasis. AMPD1 regulates cellular AMP levels [30]. AMP activates AMP kinase, an enzyme that modulates cellular energy and insulin action [30]. Paraoxonase 1 is cardioprotective due to its antioxidant activity associated with high-density lipoprotein [31] and is hypothesized to be protective against diabetes [32]. Both prenatal [33], [34], [35] and postnatal [9], [36], [37] common environment could influence glucose homeostasis and atherosclerotic risk factors in adulthood. For example, a study of the Dutch Famine in 1944–1945 demonstrated that exposure to famine during gestation resulted in increases in coronary heart disease and its risk factors in adulthood, including impaired glucose tolerance [38]. Mitochondria are critical for glucose metabolism, and its dysfunction caused by intrauterine environment was hypothesized as a fatal programming mechanism [34]. Mitochondrial dysfunction is considered to contribute to disturbed glucose homeostasis in adulthood [34], which, in turn, promotes atherosclerosis through glycation, peroxisome proliferator-activated receptors, oxidative stress, inflammation, and renin-angiotensin system dysfunction [11], [38]. A twin migration study suggested that endothelial function [10], which was associated with glucose homeostasis [39], might link postnatal common environment, including lifestyle, psychological, cultural and social factors [9], to atherosclerosis. It is biologically possible that common environment modulates genes through epigenetic mechanisms without changing genomic sequence. We believe that such an alternative mechanism falls within the broad concept of common environmental effect.

### Clinical and Preventive Implications

A review paper [40] discussed the conflicting findings on the influence of intensive glucose control with targeted glycated hemoglobin on the long-term macrovascular events and death risk from cardiovascular disease and all causes. This review [40] included three recent large-scale randomized trials among type 2 diabetic patients [41], [42], [43], one follow-up study after the intervention trials among type 2 diabetic patients [44], and one after-trial follow-up study among type 1 diabetic patients [45]. Our findings suggest that potential confounding from unknown or unmeasured common factors might be an explanation for the conflicting findings.

With regards to clinical implications, our findings imply that a treatment targeting not only hyperglycemia but also other coronary heart disease risks should be encouraged rather than solely targeting hyperglycemia to reduce the long-term risk of death from coronary heart disease. From the perspective of prevention, identification/specification of familial factors and social/cultural common factors in future research may facilitate the development of new strategies to improve survival from coronary heart disease.

### Limitations and Strengths

There were some limitations of this study. We used data from the 38-year follow-up period. Like other prospective studies, changes in other risk factors and treatment might occur during the follow-up period, which in turn might cause differential mortality across the baseline-defined groups. These changes would likely not have altered our findings. We were able to repeat the previous findings from prior prospective studies [1], [4], [5], [6] in our cohort via overall associations. Clark et al. demonstrated that selective mortality only explained the increased attenuation to a small degree in the 30-year follow-up Framingham Heart Study [46]. We showed the association was relatively stable rather than attenuated over 20 to 38-year follow-up. We did not use fasting glucose data as less baseline data were available. However, ppGlucose was shown to be a stronger predictor of cardiovascular outcomes than fasting glucose [2], [3]. We did not control for physical activity and diet in the primary analyses, since no data on physical activity were collected at baseline, and dietary data were not collected in all research centers. However, this did not affect the comparability of our overall associations with published findings from previous studies [1], [4], [5], [6] as physical activity and diet were not directly controlled for either in these studies. Like the previous studies [4], [5], [6], we controlled for baseline body mass index, which was an indicator for both adiposity and a surrogate for the medium to long term nutritional status that reflected dietary consumption relative to physical activity. It was, thus, unlikely that lack of direct control for physical activity would materially affect our results in comparison with those of previous studies [1], [4], [5], [6]. The 1-hour and the 2-hour glucose tolerance test may differ in the subject classified as diabetic [47]. The resultant potential “misclassification” is undifferentiated with respect to the outcome and would attenuate the association. However, this was unlikely to be a problem in our study since our findings were roughly similar to those found using 2-hour glucose tolerance test [6], and we obtained similar results between inclusion and exclusion of twins with baseline diabetes. Our subjects were white male twins. Although co-twin analyses controlled for genes, caution should still be taken in generalizing our findings to females and other racial groups. Our sample of veteran twins was a low risk cohort and findings from this study might not be generalized to a higher risk population. However, our study should encourage performing long-term follow-up studies in different cohorts.

Our study has several advantages over previous studies [1], [4], [5], [6]. Compared to traditional observational epidemiologic studies, the twin study design and our modeling strategy enabled simultaneous evaluation of associations free of shared genes and common environment and the role of common factors in the association. In view of epidemiology, the co-twin design naturally controls for age, cohort, and period effects. By considering ppGlucose as a continuous variable instead of categorized ppGlucose, we had an increased number of discordant twin pairs for ppGlucose and greater statistical power. Our data were collected through physical examination and the face-to-face interview using well-established Framingham Heart Study protocols which minimized measurement errors compared to self-reported data. The NHLBI Twin Study is the longest prospective twin study that was originally designed for the elucidation of genetic and environmental factors in cardiovascular diseases in the United States of America.

## Conclusion

We found that associations between ppGlucose and the risk of death from coronary heart disease, cardiovascular diseases, and all causes, independent of traditional risk factors, were largely explained by factors shared between co-twins, including familial factors. Within-pair effects cannot be absolutely eliminated. Identification of factors that are uniquely shared between dizygotic but not monozygotic co-twins may help specifying common factors. It is implied that the treatment/prevention targeting of not only hyperglycemia but also other coronary heart disease risk factors should be encouraged.

## Supporting Information

Table S1 **Aged-adjusted hazard ratios and 95% confidence intervals for specific- and all causes of death during 20 and 30 years of follow-up in the NHLBI Twin Study of nondiabetic male twins aged 42 to 55 years at baseline.**
(DOC)

Text S1 **Interpretation of between-pair effects with an example.**
(DOCX)
