# Supplementary material for: The Association between Postload Plasma Glucose Levels and 38-Year Mortality Risk of Coronary Heart Disease: The Prospective NHLBI Twin Study
Source: PLoS One. 2013 Jul 19;8(7):e69332. doi: 10.1371/journal.pone.0069332 (PMC3716604; doi:10.1371/journal.pone.0069332)
Supplement: Table S1 — Aged-adjusted hazard ratios and 95% confidence intervals for specific- and all causes of death during 20 and 30 years of follow-up in the NHLBI Twin Study of nondiabetic male twins aged 42 to 55 years at baseline. (DOC) [file pone.0069332.s001.doc]

**Table S1. Age-adjusted hazard ratios and 95% confidence intervals for specific- and all causes of death during 20 and 30 years of follow-up in the NHLBI Twin Study of nondiabetic male twins aged 42 to 55 years at baseline*.**

| **Death Cause** | **No. of Death** | | **Overall Effects** | **Within-Pair Effects** | **Between-Pair Effects** | **Interaction between**  **Within-Pair Effect and Zygosity** |
| --- | --- | --- | --- | --- | --- | --- |
| **20-Year follow-up (total person-years = 17032)** | | | |  |  |  |
| Coronary Heart Disease | | 29 | 1.20 (0.95, 1.49) | 1.01 (0.73, 1.39) | 1.27 (1.001, 1.60) | 0.12 |
| Cardiovascular Disease | | 49 | 1.14 (0.97, 1.34) | 1.02 (0.80, 1.30) | 1.18 (0.99, 1.40) | 0.45 |
| All-Causes | | 143 | 1.11 (0.99, 1.24) | 1.07 (0.90, 1.27) | 1.12 (0.99, 1.27) | 0.94 |
| **30-Year follow-up (total person-years = 23517)** | | | |  |  |  |
| Coronary Heart Disease | | 72 | 1.22 (1.07, 1.3) | 1.07 (0.89, 1.28) | 1.27 (1.11, 1.45) | 0.31 |
| Cardiovascular Disease | | 128 | 1.14 (1.05, 1.25) | 1.06 (0.93, 1.20) | 1.17 (1.07, 1.29) | 0.59 |
| All-Causes | | 367 | 1.07 (1.01, 1.13) | 1.02 (0.94, 1.11) | 1.09 (1.03, 1.16) | 0.43 |

*Hazard ratio was age adjusted. Age adjustment was applied to between-pair effects and overall effects.
