# Supplementary material for: The Association between Postload Plasma Glucose Levels and 38-Year Mortality Risk of Coronary Heart Disease: The Prospective NHLBI Twin Study
Source: PLoS One. 2013 Jul 19;8(7):e69332. doi: 10.1371/journal.pone.0069332 (PMC3716604; doi:10.1371/journal.pone.0069332)
Supplement: Text S1 — Interpretation of between-pair effects with an example. (DOCX) [file pone.0069332.s002.docx]

**Text S1. Interpretation of between-pair effects with an example.**

Between-pair effects were interpreted as the expected difference in the log hazard ratio between any two unrelated twin subjects with the same deviation of ppGlucose from the mean of his twin pair but one-unit difference in the pair mean [[1](#_ENREF_1)]. This could be illustrated: twin pairs A and B had the same within-pair difference in ppGlucose but the mean ppGlucose of twin pair A was one-unit higher than that of twin pair B. If we select one subject from each twin pair who had the higher ppGlucose, between-pair effects reflect the expected relative risk comparing the subject from twin pair A with that from twin pair B. For between-pair effects, ppGlucose was considered as a surrogate for influences of genes and environment shared between co-twins in relation to ppGlucose.

**References for Supplemental Information**

1. Carlin JB, Gurrin LC, Sterne JA, Morley R, Dwyer T (2005) Regression models for twin studies: a critical review. Int J Epidemiol 34: 1089-1099.
